# Gold-silver alloy nanoshells: a new candidate for nanotherapeutics and diagnostics

**DOI:** 10.1186/1556-276X-6-554

**Published:** 2011-10-13

**Authors:** Dana E Gheorghe, Lili Cui, Christof Karmonik, Audrius Brazdeikis, Jose M Penaloza, Joseph K Young, Rebekah A Drezek, Malavosklish Bikram

**Affiliations:** 1 Department of Pharmacological & Pharmaceutical Sciences, College of Pharmacy, University of Houston, Texas Medical Center Campus, 1441 Moursund St., Houston, TX 77030, USA; 2 The Methodist Hospital, 6565 Fannin, Houston, TX 77030, USA; 3 Department of Physics and Texas Center for Superconductivity, University of Houston, 4800 Calhoun Road, Houston, TX 77004, USA; 4 Department of Electrical and Computer Engineering, Rice University, 6100 Main Street, MS-366, Houston, TX 77005, USA; 5 Department of Bioengineering, Rice University, 6100 Main Street, MS-142, Houston, TX 77005, USA

## Abstract

We have developed novel gold-silver alloy nanoshells as magnetic resonance imaging (MRI) dual *T*_1 _(positive) and *T*_2 _(negative) contrast agents as an alternative to typical gadolinium (Gd)-based contrast agents. Specifically, we have doped iron oxide nanoparticles with Gd ions and sequestered the ions within the core by coating the nanoparticles with an alloy of gold and silver. Thus, these nanoparticles are very innovative and have the potential to overcome toxicities related to renal clearance of contrast agents such as nephrogenic systemic fibrosis. The morphology of the attained nanoparticles was characterized by XRD which demonstrated the successful incorporation of Gd(III) ions into the structure of the magnetite, with no major alterations of the spinel structure, as well as the growth of the gold-silver alloy shells. This was supported by TEM, ICP-AES, and SEM/EDS data. The nanoshells showed a saturation magnetization of 38 emu/g because of the presence of Gd ions within the crystalline structure with *r*_1 _and *r*_2 _values of 0.0119 and 0.9229 mL mg^-1 ^s^-1^, respectively (Au:Ag alloy = 1:1). *T*_1_- and *T*_2_-weighted images of the nanoshells showed that these agents can both increase the surrounding water proton signals in the *T*_1_-weighted image and reduce the signal in *T*_2_-weighted images. The as-synthesized nanoparticles exhibited strong absorption in the range of 600-800 nm, their optical properties being strongly dependent upon the thickness of the gold-silver alloy shell. Thus, these nanoshells have the potential to be utilized for tumor cell ablation because of their absorption as well as an imaging agent.

## 1. Introduction

The search for new composite systems consisting of a wide range of metal and semiconductor core materials with an outer inert shell has led to the discovery of novel nanoparticles with a broad range of biomedical applications in areas such as tissue engineering, gene and drug delivery, photo-thermal therapy, cell tracking, and storage systems [1-3]. Among this broad area of nanometer-sized systems, iron oxide nanocores have gained special attention because of their unique physical properties in which their size, morphology, composition, and surface chemistry can be tailored to many biological and biomedical applications [4,5]. Tantamount to their physical versatility, these nanocores possess unique magnetic properties that facilitate proton relaxation within specific tissues, which thus make them suitable as *T*_2 _contrast agents for magnetic resonance imaging (MRI) [6,7]. Typical iron oxides that have extensively been explored for MRI applications include magnetite, Fe_3_O_4_, with alternating lattices of Fe(II) and Fe(III) ions or maghemite, γ-Fe_2_O_3_, in which the lattice structure consist of Fe(III) ions only [8]. However, despite their widespread use in the biomedical field, an open challenge for using iron oxide nanoparticles as contrast agents is to improve their magnetic properties, which in turn would lead to higher imaging sensitivity. This can be done by doping them with transition metal ions, in particular lanthanides which have distinctive magnetic and optical properties, associated with their electronic configuration [9,10].

However, apart from their magnetic properties, these nanocores are unstable both in air and in solution. Iron oxide typically forms aggregates in solution and undergoes oxidation in air [11,12]. In addition, even though iron oxide nanoparticles are good magnetic materials, the cores are susceptible to corrosion in the presence of water [13,14] and are more reactive in bulk materials because of their high surface-to-volume ratio [15]. Also, aside from their physical instability and relative biocompatibility [16,17], iron oxide nanocores have been shown to be toxic [18]. However, these hurdles can be overcome by coating the cores with outer shells, such as inorganic oxides (silica) [19,20], inert metals (silver, gold, or gold alloys) [11,21-23], or bioactive macromolecules such as liposomes and micelles, which can encapsulate a numerous amount of nanoparticles within the structures that then impart an inert physical property to the cores in biological media [2,24-26]. Subsequent surface functionalization of the nanocomposite materials with hydrophilic polymers such as poly(ethylene glycols) (PEGs) [27-30] and dextrans [31,32] can further increase the physical stability of the nanoparticles particularly in solution. In addition, the coating of the magnetic nanoparticles with inert metals, such as gold, is extremely attractive because the nanocomposite system is resistant to corrosion in biological conditions and can decrease the toxicity of the cores [33-35].

Moreover, coating of these doped cores with an outer inert shell can sequester toxic metals such as gadolinium within the core of the nanoparticles [12]. Gd(III) ions have to be combined with carrier molecules because of their extreme toxicities, which are strongly associated with a systemic fibrosing disorder that is referred to as nephrogenic systemic fibrosis (NSF) in patients with kidney diseases [36,37]. The Gd ions are toxic because their ionic radius is almost equal to that of divalent calcium ions, which enable Gd to compete with biological systems including enzymes with a higher binding affinity to alter the kinetics of their systems [38]. In addition, Gd ions are inorganic blockers of voltage-gated calcium channels [39]. Hence, the development of a contrast agent that could completely sequester Gd within the core of the nanoparticle, thereby preventing its release into the body would highly be beneficial as a safe and effective alternative to typical Gd-based contrast agents.

Currently, there is an extensive development of monometallic nanoshells with minor attention given to nanosystems consisting of bimetallic and trimetallic alloy shells [40-43]. Recent research in this area has shown that gold-silver alloy nanoshells might have additional biomedical applications (e.g., cancer screening), because of their distinct optical properties ranging from visible to the near-IR wavelength region [44]. Our exploratory research aimed at preparing new biocompatible nanoshells for MR imaging that could overcome NSF and can also be utilized for laser ablation of tumor cells has led to the synthesis of novel Gd-doped magnetite cores, Gd:Fe_3_O_4_, covered with a gold-silver alloy shell.

## 2. Experimental method

### 2.1. Materials and synthesis

**Figure 1:**
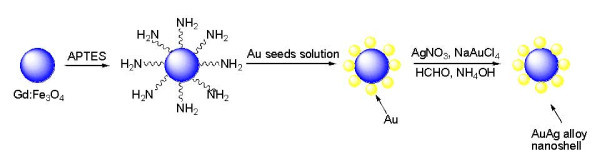
**Schematic illustration of the preparation of the Gd-doped iron oxide nanoparticles with gold-silver alloy nanoshells**.

FeCl_2 _4H_2_O (99%), FeCl_3 _6H_2_O (97%), GdCl_3 _(99.9%), AgNO_3_, HCl (37%), NaAuCl_4 _(99%), HNO_3 _(70%), NaOH (98%), NH_4_OH (30%), 3-aminopropyltriethoxysilane (APTES), formaldehyde, and tetrakis(hydroxymethyl)phosphonium (THPC) were purchased from Sigma-Aldrich and used as-received unless otherwise stated. The magnetite nanoparticle cores were doped with a molar ratio of 10% Gd(III) ions that were prepared by a coprecipitation reaction of Fe(III), Fe(II), and Gd(III) salts in NaOH solution at 65°C. The surface of the nanocores was functionalized with -NH_2 _groups by the addition of 3-aminopropyltriethoxysilane (APTES) [45]. This resulted in surface-terminated -NH_2 _groups of APTES that were available for covalent coordination to a transition metal through the lone pair of nitrogen. As previously reported in basic medium [46,47], the terminal amine groups of the amine-functionalized nanocores act as anchoring sites for the gold nanoparticles used in the seeding step by forming covalent Au-N bonds (gold metal being well known for its affinity for binding with amine or thiol terminal groups of organic molecules [48]). A gold seed solution was then prepared by reducing NaAuCl_4 _with tetrakis(hydroxylmethyl)phosphonium chloride (THPC), as reported by Duff [49,50]. The gold-seeded method was utilized since the gold nanoparticles can be used as nucleation sites for the growth of a mono or bimetallic shell. Moreover, this method enabled control over shell thickness and composition of the synthesized nanoshells [43]. Finally, the gold-seeded amine-functionalized nanocores were used as nuclei to build up a gold-silver alloy nanoshell formed as a result of a redox reaction, which occurred in the presence of formaldehyde as a reducing agent, when the THPC gold-seeded Gd-doped iron oxide nanoparticles were added to a basic solution of Au(III) and Ag(I) ions corresponding to molar ratios of Au:Ag = 1:1 and 5:1, respectively. Au(III) and Ag(I) ions were simultaneously reduced to metallic gold and silver, forming the alloy-based shell (see Figure 1).

### 2.2 Characterization and measurements

Powder X-ray diffraction analysis was used for the analysis of the synthesized samples. Data were collected at room temperature using a PANanalytical X'Pert PRO diffractometer equipped with Cu Kα radiation (λ = 1.5406 Å). The samples were ground using an agate mortar and pestle to a suitable powder sample. The powder was then placed on a zero background sample holder. The 2θ range was 5-120° with a scan rate of 6.4°/min. Data compilation and analysis were processed using the Diffractometer Management System software package which included a JCPDS powder diffraction database [51]. The composition of the samples was analyzed using a sequential Thermo Jarrell Ash Atomscan 25 inductively coupled plasma (ICP) emission spectrometer. Measurements were made using the following selected lines: 230.562 for Fe, 328.068 for Ag, 242.795 for Au, and 342.347 for Gd. The instrument drift was corrected by running a quality control solution every two to three analyses. The nanoparticles were dissolved in aqua regia and evaporated to dryness. The resulted residues were dissolved in 10% nitric acid and diluted to concentrations in the range of 1-50 ppm for analysis. Standard solutions with concentrations ranging from 0 to 50 ppm were used for calibration curves. Chemical analyses of the samples by means of SEM/EDS were performed on a Jeol JSM 8330 F scanning electron microscope equipped with an energy-dispersive spectrometer (EDS) by means of 15 kV accelerating voltage and 12 μA emission current from a cold-field emission gun. Small volumes of water-dispersed nanoparticles were deposited on a copper disk and allowed to evaporate at room temperature. The copper disk was subsequently attached to a brass sample holder using carbon tape. All the analyses were carried out at approximately 4 × 10^-4 ^Pa. Elemental analysis was used for all our samples. These results complemented the results from XRD and ICP-AES analyses. The size and morphology of the nanoparticles were determined with transmission electron microscopy (TEM) on a JEOL JEM-2000 FX instrument. Statistical analysis and average size distribution of the particles were determined by evaluating a minimum of 100 nanoparticles per sample. The mean standard deviation σ, defined as σ = ((1/(*N *- 1))*Σ*_i_*(*x_i _*- *x*)^2^)^1/2^, was also determined for each size distribution. The nanoparticles were dispersed in water and cast on a carbon-coated grid (Pelco^® ^No. 160). The sample was allowed to settle on the grid for approximately 1 min in a humidified atmosphere. The carbon-coated grid was then washed with water, followed by another addition of nanoparticles onto the carbon-coated grid. The grids were then left for another 1 min before gently removing excess liquid with a filter paper. The absorption of the nanoparticles in the ultraviolet-visible-near infrared region (UV-Vis-NIR) was determined with a Beckman Coulter DU 800 spectrophotometer using a 0.1-mm quartz cuvette. The spectra were collected in the range of 190-1100 nm at a resolution of 0.5 nm, and the concentration of the nanoparticles was adjusted so that the maximum absorbance did not exceed 1 absorbance unit. The magnetic susceptibility of the synthesized nanoparticles was measured by a superconducting quantum interference device magnetometer (Quantum Design MPMS). The saturation magnetization measurements were performed at 300 and 5 K. Once the desired measurement temperature was reached at zero field, field cycling from 5 to -5 T back and forth was applied followed by data collection for each M(H) measurement curve. In addition, the zero-field-cooled (ZFC) and field-cooled (FC) measurements were performed by cooling the sample from 300 to 5 K at zero field and then applying 200 Oe field for the warming up scan. Longitudinal and traverse relaxation times were measured in a 1.5-T magnet on a Siemens Avanto, full body clinical scanner (Siemens AG Healthcare) at 37°C. The coil used was standard 12 channel head coil and the samples prepared were dispersed in 1% agarose gel at the following nanoparticle concentrations: 0.005, 0.01, 0.02, 0.03, and 0.04 mg/mL. In brief, solutions were solidified in test tubes of 10 mm diameter, placed in a sample holder, and simultaneously imaged together with calibration samples of copper sulfate (CuSO_4 _H_2_O) prepared in a similar fashion for the following concentrations: 6.54, 5.872, 5.538, 5.204, 4.87, 4.536, 3.868, 3.2, 2.08, 0.96, and 0.52 mg/mL. Fast spin echo images (echo train length 16, acquisition matrix = 256 × 128, field of view (FOV) = 220 × 110 mm resolution in an isotropic in-plane resolution of 0.86 mm, slice thickness 5 mm) were acquired in two series. In series 1, the echo time was varied from 10 to 160 ms (in increments of 10 ms) with fixed repetition time (TR = 3000 ms). From the signal intensity curves of these images, the spin-spin relaxation time *T*_2 _was determined for each concentration. In series 2, images were acquired for the following TRs: 170, 350, 700, 1000, 2000, 4000, and 5000 ms while keeping the echo time constant at minimum (TE = 9.9 ms). Gray scale values from the MRI images acquired at different TR and TE times were analyzed as follows: signal intensities were determined as average over regions of interest within the test tube (using Image J, NIH) avoiding image voxel at the test tube wall to minimize partial volume averages. The nonlinear Levenberg-Marquardt fitting method was then used to the fit standard equations for MRI signal intensity for spin echo sequences [52] to both the signal intensities at different TE (series 1) to determine *T*_2 _and to the signal intensities at different TR to determine *T*_1_. These fits were performed for the different concentrations of the Au/Ag alloy-coated nanoparticles, the commercial Omniscan^®^, and the CuSO_4 _H_2_O standards. Relaxivities, *r*_1 _and *r*_2_, were then determined using linear regression analysis assuming a linear relationship between relaxation times *T*_1 _and *T*_2 _and concentration. For Omniscan^® ^and CuSO_4 _H_2_O, relaxivities were compared to literature values.

## 3. Results and discussion

**Figure 2:**
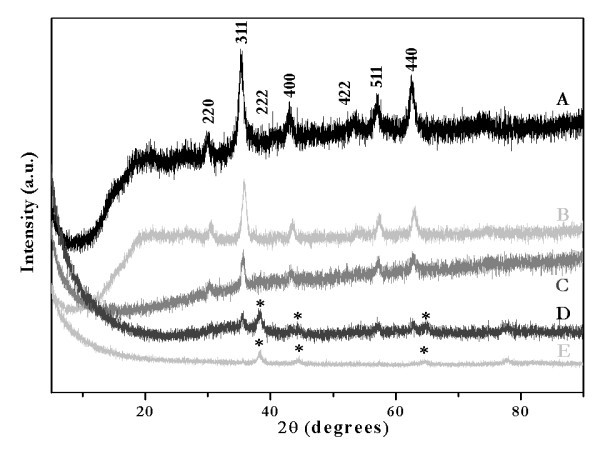
XRD patterns of Fe_3_O_4 _(A), Gd:Fe_3_O_4 _(B), gold-seeded Gd:Fe_3_O_4 _(C) and gold-silver alloy (*) deposited on gold-seeded Gd:Fe_3_O_4 _nanocores, Au:Ag = 1:1 (D) and Au:Ag = 5:1 (E). Miller indices are indicated for Fe_3_O_4_.

Previously, experimental data reported for an active bimetallic Au-Ag nanoparticle system on inert supports, such as silica, as well as density functional theory calculations performed on selected model clusters proposed for the Au-Ag bimetallic system, indicated that Au-Ag alloy shells formed through adsorption of positively charged Ag^+ ^ions on negatively charged Au^3+ ^ions followed by their reduction with NaBH_4 _[53]. These interactions take place via electrostatic attractions as well as strong Au-Ag metallic interactions in which the later was found to be more predominant. Elemental analysis of the as-synthesized nanoshells by ICP-AES (Table 1) confirmed the presence of Gd ions, which gave molar ratios of Gd:Fe = 0.17:1 and 0.11:1 for the theoretical molar ratios of Au:Ag = 1:1 and 5:1, respectively. Thus, it was estimated that 17 and 11% of Gd(III) ions used in the preparation of the doped nanocores were incorporated into the final Au:Ag = 1:1 and 5:1 nanoparticles, respectively, which gave core compositions of Gd_0.17_Fe_2.83_O_4 _and Gd_0.11_Fe_2.89_O_4_. The crystallinity and structure of the Gd:Fe_3_O_4 _nanocores, as well as the gold-silver alloy nanoshells, were studied by means of XRD. As shown in Figure 2B, the X-ray powder diffraction pattern confirmed the formation of a single phase of the Gd-doped magnetite nanocores, which had the cubic spinel structure of the well-studied Fe_3_O_4 _(Figure 2A) [54]. The broad peak at approximately 20° in the diffraction powder pattern corresponds to the sample holder because of difficulties in the preparation of well-packed samples.

**Table 1:** Compositions obtained by ICP-AES for the analysis of Fe_3_O_4_, Gd:Fe_3_O_4_, Au-seeded functionalized Gd:Fe_3_O_4_, and gold-silver alloy nanoshells on gold-seeded amine-functionalized Gd:Fe_3_O_4_

Sample	Gd ppm	Fe ppm	Au ppm	Ag ppm	Gd:Fe	Au:Ag
Fe_3_O_4_	0	70.1	0	0	-	-
Gd: Fe_3_O_4_	11.6	57.7	0	0	0.2	-
Au-seeded Gd: Fe_3_O_4_	12.2	62.6	1	0	0.2	-
Au-Ag alloy = 1:1 nanoshells on Au-seeded amine-functionalized Gd: Fe_3_O_4_	7.0	40.4	6.8	19.5	0.2	0.4
Au-Ag alloy = 5:1 nanoshells on Au-seeded amine-functionalized Gd: Fe_3_O_4_	7.7	67.6	24.0	4.5	0.1	5.3

There was no significant change in the X-ray powder pattern of the Gd:Fe_3_O_4 _nanoparticles compared to the parent compound, Fe_3_O_4_, which was prepared as a reference by a coprecipitation reaction of Fe(III) and Fe(II) in NaOH at 65°C (see Figure 2A, B). These results are consistent with nanoparticles previously synthesized by the coprecipitation of the Fe(II), Fe(III), and Gd(III) salts in the same basic solution, which did not result in a mixed oxide system, Gd_2_O_3_-Fe_3_O_4_, but a doped Gd:Fe_3_O_4 _[55]. The powder pattern of the as-synthesized nanoparticles indicated a smaller unit cell for the doped nanocores that was confirmed by the shift of the diffraction peaks characterized by Miller indices shown in Figure 2A (220, 311, 222, 400, 422, 511, and 440) toward bigger Bragg angles. Indexing of the powder patterns presented in Figure 2A, B indicated for Gd:Fe_3_O_4 _a cubic unit cell with lattice parameter *a *= 8.308 Å, which is approximately 0.8% smaller than the unit cell of the parent compound, Fe_3_O_4_, *a *= 8.373 Å, respectively (reported data for Fe_3_O_4_: cubic unit cell, space group *Fd-3m, a *= 8.384 Å) [56]. It was also suggested that in such doped system, the Gd(III) ions might occupy the octahedral sites [57]. The average particle size of the nanocores was estimated by applying the Debye-Scherrer model, written as *d *= 0.9λ/ß*_d_*cosθ, where *d *is the crystallite diameter, λ = 1.540562 Å for Cu Kα line, ß*_d _*is the full width at half maximum of the strongest reflection peak, and θ is the corresponding Bragg angle [58]. This formula was applied for the (311) reflection in the powder pattern shown in Figure 2B in which an average crystallite size of 3.5 nm for the nanocores was calculated. The gold seeding step of the Gd:Fe_3_O_4 _nanocores had as a result--a decrease in the intensities characteristic to the doped spinel structure (Figure 2C). The X-ray powder diffraction was also used as a tool to verify the formation of the gold-silver alloy nanoshells on the gold-seeded Gd:Fe_3_O_4 _nanocores. As can be seen from Figure 2D, apart from the weak peaks which corresponded to the (311), (422), (511), and (440) reflections of the Gd:Fe_3_O_4_, three extra peaks positioned at 2θ values of 38.4°, 44.6°, and 64.7° that corresponded to the gold-silver alloy (molar ratio Au:Ag = 1:1) were observed. In Figure 2E, peaks corresponding to the gold-silver alloy (Au:Ag = 5:1) only are observed. These data indicated the formation of a layer of gold-silver alloy on the nanocores and not the formation of discrete gold and silver nanoparticles, which would have shown six individual peaks: 38.2°, 44.4°, and 64.6° corresponding to (111), (200), and (220) planes for gold [59] and 38.4°, 44.3°, and 64.7° corresponding to (111), (200), and (220) planes for silver [60], respectively. Moreover, the XRD patterns of gold and silver are completely overlapped in Figure 1D, E; therefore, the gold-silver alloy cannot be distinguished from the powder patterns of gold and silver as monometallic phases [61]. In addition, the absence of any diffraction peaks for Gd-doped magnetite cores in Figure 2E is because of the heavy atom effect from gold; it was observed that gold shells dominate the powder pattern because of its high electron density, which is consistent with previously reported data [58]. Our observation is consistent with data reported for gold nanoshells deposited on magnetite cores by others [62-64], as well as with ICP-AES results (Table 1) which indicated a molar ratio of Au:Ag = 0.4 (theoretical ratio 1:1) and 5.3, respectively (theoretical ratio 5:1). In addition, Liu et al. showed that the powder pattern of gold-silver alloy shells deposited on silica cores exhibited only the peaks characteristic to the gold or silver monometallic phase, whereas the peaks corresponding to the silica support were found to be absent [53]. Thus, our findings from the TEM, ICP-AES, and SEM/EDS data together with previously published one suggested that the Gd:Fe_3_O_4 _nanocores were completely covered with the gold-silver alloy and hence the formation of the nanoshells. Consistent with these findings was the optical absorption data for the nanoshells, which exhibited only one plasmon band that further supported the suggested nanoshell structure (discussed later). Therefore, we have shown that the reduction of gold and silver salts in the presence of gold-seeded Gd:Fe_3_O_4 _cores successfully led to the formation of gold-silver alloy nanoshells. However, the gold-silver shell is very thin when Au:Ag = 1:1 since the diffraction from gold and silver does not dominate the powder pattern, but a decrease of the intensity of the reflection peaks for the Gd:Fe_3_O_4 _nanocores was observed (Figure 1D) [1,64]. As the ratio of Au:Ag was increased to 5:1, the reflections of gold and silver started to dominate the powder pattern and most of the characteristic peaks of the nanocore disappeared (Figure 2E).

**Figure 3:**
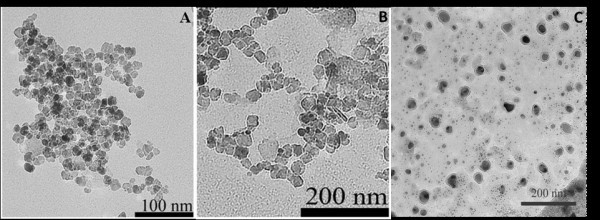
**Representative TEM images of as-synthesized **(A) **Gd:Fe_3_O_4 _nanocores; **(B) **gold-silver alloy (Au:Ag = 1:1) nanoparticles; and gold-silver alloy (Au:Ag = 5:1) nanoparticles**.

**Figure 4:**
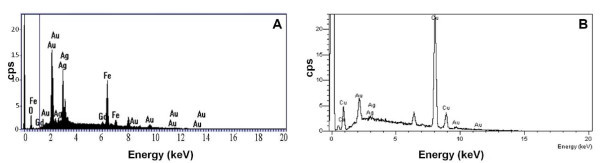
**SEM/EDS spectra of as-synthesized gold-silver alloy nanoshells**. Theoretical ratio of **(A) **Au:Ag = 1:1, experimental ratio Au:Ag = 3:1, and **(B) **Au:Ag = 5:1, experimental ratio Au:Ag = 3.6:1.

Statistical analysis and average size distribution of the particles were determined by evaluating a minimum of 100 nanoparticles per sample set of Gd:Fe_3_O_4_, Au:Ag = 1:1, and Au:Ag = 5:1 nanoparticles. A representative set of TEM micrographs of the Gd:Fe_3_O_4 _nanoparticles, gold-seeded Gd:Fe_3_O_4_, and gold-silver alloy nanoshells (Au:Ag = 1:1 and 5:1) used for characterization in all studies is shown in Figure 3. There are two major findings from the morphological comparison of the images. First, the nanoparticles exhibited ovoid features with a high degree of crystallinity and an average size diameter of 8.0 ± 2.8 nm for the Gd:Fe_3_O_4 _nanocores, 8.2 ± 0.4 nm for the gold-silver alloy nanoshells (Au:Ag = 1:1), and 18.6 ± 3.6 nm for the gold-silver alloy nanoshells (Au:Ag = 5:1) (Figure 3A-C, respectively). In addition, the nanoshells corresponding to Au:Ag = 5:1 showed a much lighter coat surrounding the dark Gd:Fe_3_O_4 _core (Figure 3C) (average thickness of the coating = 5.0 ± 2.8 nm) compared to the gold-silver alloy nanoshells at a 1:1 ratio (Figure 3B). Additional TEM micrographs of this sample are shown in "Figure S1 in Additional file 1". In addition, these nanoshells are unique since the alloy shell surrounding the core (Figure 3C) was clearly observed in the TEM images even though it has previously been considered impossible to observe an additional metal shell deposited on the surface of magnetite nanocores [65]. Most probably the lighter color was because of the presence of silver metal within the alloy shell. This observation was consistent with published reports for gold-palladium shells deposited on silica nanocores [43]. In these nanoshells, the lighter color of the surrounding shells around the cores was attributed to the gold-palladium alloy [43]. However, in the case of Au:Ag = 1:1 ratio the alloy shell was not apparently visible (Figure 3B) because of much thinner shell. In this case, the formation of the gold-silver shell was clearly confirmed by XRD (see Figure 2D) as well as semi-qualitative SEM/EDS analysis (see Figure 4A,B). Moreover, the measurement of the surface plasmon resonance band (discussed later) of these nanoparticles provided an indirect piece of evidence supporting the formation of the alloy nanoshells. Another important observation for the Au:Ag = 1:1 ratio nanoshells was that the dispersion of the nanoparticles was not as pronounced as in the case of the 5:1 ratio when the shell was clearly visible and implicitly thicker (see "Figures S1 and S2 in Additional file 1").

**Figure 5:**
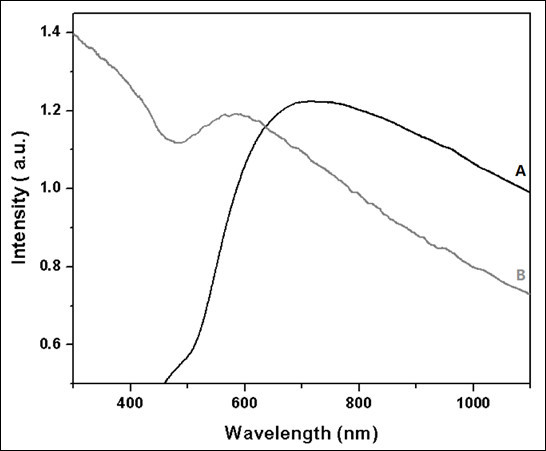
**UV-Vis-NIR spectra of as-synthesized gold-silver alloy nanoshells deposited on gold-seeded Gd:Fe_3_O_4 _nanocores for **(A) **Au:Ag = 1:1 and **(B) **Au:Ag = 5:1 nanoparticles**.

The optical properties of the as-synthesized nanoshells were studied with UV-Vis-NIR spectroscopy over the wavelength range of 190-1100 nm. Systematic studies with reaction times and absorption of nanoparticles (data not shown) indicated that the optimum reaction time for the deposition of the gold-silver alloy was 7 days (see "Figure S3 in Additional file 1"). This was further confirmed by high absorption intensity for the 7 days reaction time; based on Beer-Lambert law this might indicate the complete formation of the alloy nanoshells [64]. The UV-Vis-NIR spectra of the as-synthesized alloy nanoshells for 7 days reaction time for both Au:Ag = 1:1 and 5:1 ratios, respectively, are depicted in Figure 5. As can be seen, maximum absorption peaks were observed at approximately 700 nm for 1:1 ratio (Figure 5A) and approximately 600 nm for 5:1 ratio (Figure 5B), which fell in between those of silver nanoparticles (420 nm) and gold nanoshells (approximately 800 nm) [65]. These data also confirmed that the mixture of the reduced gold and silver ions (Au:Ag = 5:1) in the alloy did not produce two distinctive bands in the spectrum and that the surface plasmon resonance property of the synthesized nanoshells was dependent on the thickness, as reported in the literature [66-70]. Furthermore, when the ratio Au:Ag was 1:1, two distinctive plasmon bands were observed in the UV-Vis-NIR spectra for 1, 7, and 14 days reaction time (see "Figure S3 in Additional file 1"); for 7 days reaction time one band was centered at 480 nm and the second one at 700 nm, the first band being attributed to the initial stage of the Au-Ag nanoshell grow [53,65]. The absence of the second plasmon band for a ratio of Au:Ag = 5:1 may be because of a higher concentration of gold in the gold-silver alloy. The 1:1 ratio spectra (Figure 5A) exhibit a broad plasmon band that extends out toward 1100 nm. It is believed that the observed spectral shoulder that extends out from 850 toward 1100 nm can be attributed to particle aggregation. Nonetheless, the bulk of the sample consist of individual particles where the width of the extinction peak, centered at 700 nm, can be attributed to a combination of line broadening and inhomogeneous broadening where inhomogeneous broadening is a result of the nanoparticle size and shape distributions and makes up the bulk of the peak width. This inhomogeneous broadening effect was observed and reported by others for simular metallic shell configurations [69-74]. Thus, our findings are in a good agreement with reported surface plasmon resonance data for gold-silver alloy nanoshells when the absorption was tuned from 430 to 780 nm. Since we were interested in using these nanoparticles for both MR imaging and as a therapeutic tool for thermal ablation, we selected the gold-silver alloy at the 1:1 ratio for subsequent analysis and physical characterization.

**Figure 6:**
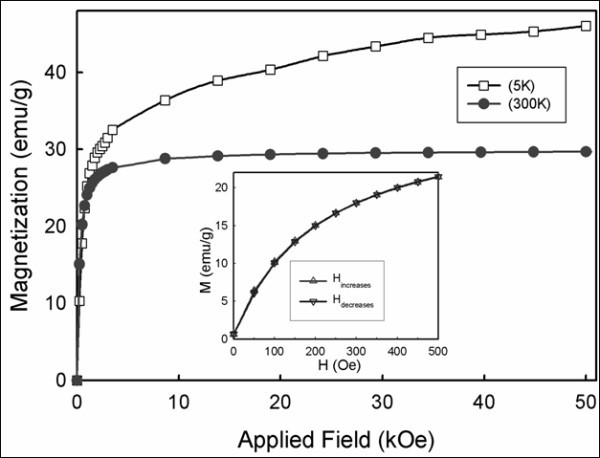
**Magnetization versus applied magnetic field plots for the Au-Ag alloy (1:1) nanoshells sample at 300 and 5 K**. The inset shows an expanded view of the low magnetic field behavior at 300 K.

A common feature of magnetization curves *M*(*T*) is the occurrence of a maximum in ZFC curves at a temperature, associated with the blocking temperature of Gd:Fe_3_O_4 _nanocores. Analysis of the blocking temperatures (250-280 K) of the as-synthesized Au-Ag alloy = 1:1 nanoshells was found to be consistent with Stoner-Wohlfarth theory and average sizes of Gd:Fe_3_O_4 _nanocores. Moreover, the variation of the magnetization in the ZFC and FC measurements indicated a superparamagnetic behavior for the Au-Ag alloy (1:1) nanoshells despite the presence of the metallic shell. From the *M*(*H*) measurements (Figure 6), the normalized saturation magnetization at 300 K was 30 emu/g and approached 38 emu/g Gd:Fe_3_O_4 _for the Au-Ag alloy = 1:1 nanoshells. Owing to the Gd ions occupying the octahedral sites in Gd-doped Fe_3_O_4_, the magnetization value was found to be slightly reduced compared to undoped Fe_3_O_4_, consistent with observation by others [75]. Thus, the above magnetic characterization data support the use of Au-Ag alloy nanoshells as superparamagnetic MR contrast agents.

**Figure 7:**
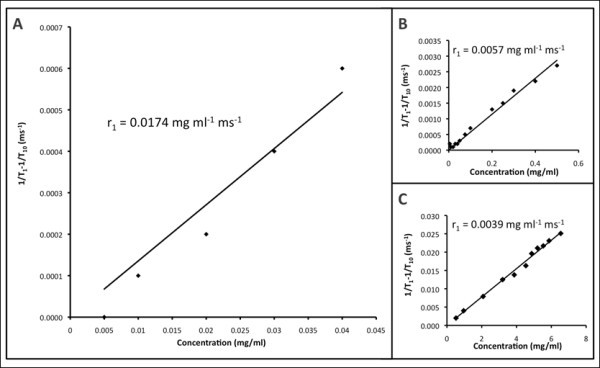
***T*_1 _relaxation rates for **(A) **Au/Ag alloy-coated nanoparticles, **(B) **commercial Omiscan^®^, and **(C) **CuSO_4 _H_2_O**.

**Figure 8:**
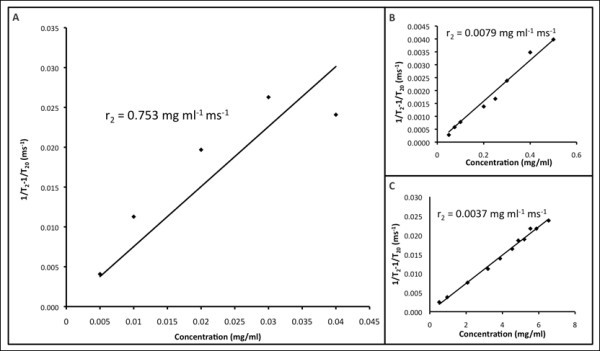
***T*_2 _relaxation rates for **(A) **Au/Ag alloy-coated nanoparticles, **(B) **commercial Omniscan^®^, and **(C) **CuSO_4 _H_2_O**.

**Figure 9:**
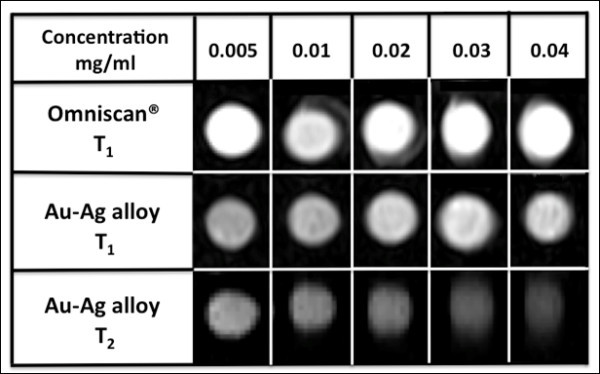
**MR images of **(A) ***T*_1 _and **(B) ***T*_2_-weighted Au/Ag alloy-coated nanoparticles at different concentrations**.

The relaxivity rates *r*_1 _(*T*_1 _relaxivity) and *r*_2 _(*T*_2 _relaxivity) were calculated from 1/*T*_1 _after subtracting 1/*T*_10 _(*T*_10 _of agarose gel from measurement: 3333.333 ms) and from 1/*T*_2 _after subtracting 1/*T*_20 _(*T*_20 _of agarose gel from measurement: 144.5 ms), respectively. The *r*_1 _and *r*_2 _values of CuSO_4 _were calculated to be 0.0039 and 0.0037 mL mg^-1 ^s^-1^, respectively (Figures 7C and 8C), which were found to be similar to values published in the literature (*r*_1_: 0.94 mM^-1 ^s^-1^) [76]. The *r*_1 _and *r*_2 _values of Omniscan^® ^were calculated to be 0.0057 and 0.0079 mL mg^-1 ^s^-1^, respectively (Figures 7B and 8B). The ratio of *r*_1 _and *r*_2 _was 0.72 which is close to the value published in the literature (4.1/4.7 = 0.87) [77]. The *r*_1 _and *r*_2 _values of the as-synthesized Au-Ag alloy = 1:1 nanoshells were found to be 0.0174 and 0.7532 mL mg^-1 ^s^-1^, respectively (Figures 7A and 8A). A nonlinearity for 1/*T*_1 _and 1/*T*_2 _at higher concentrations was noted for the as-synthesized Au-Ag alloy = 1:1 nanoshells (Figures 7A and 8B). Even though this nonlinearity of the data was unexpected it is assumed, potentially that particle clustering occurred, which effectively reduced the coordination number for the water molecules surrounding the as-synthesized nanoshells. Carrying out the above-described analysis for the linear range only, the following values for *r*_1 _and *r*_2 _were obtained: *r*_1 _= 0.0119 and *r*_2 _= 0.9229 mL mg^-1 ^s^-1^. In order to validate the potential of Au-Ag alloy nanoshells as both *T*_1 _and *T*_2 _MR contrast agents, we acquired *T*_1_- and *T*_2_-weighted images as a function of nanoshell concentration. As shown in Figure 9, the alloy-coated nanoshells produced increasingly bright images that corresponded to increasing concentrations of the nanoshells for the *T*_1_-weighted MR images, which resembled lighter images typical of *T*_1 _contrast agents rather than *T*_2 _contrast agents such as iron oxide. In addition, the *T*_2_-weigted images for the alloy-coated nanoshells produced increasingly darker images as a function of concentration that corresponds to the reduction of the MR signal characteristic of iron-oxide-based MR contrast agents. Therefore, these images support the use of Au-Ag alloy nanoshells as dual *T*_1 _and *T*_2 _contrast agents.

## 4. Conclusion

Based on previous investigations [44,64,78] as well as our experimental observations, we anticipate that the optical properties of the nanoshells can be tailored in a controlled manner by adjusting the volume of the gold seed solution as well as the amounts of gold and silver salts used as precursors in the formation of the nanoshells. Studies regarding this matter, the optimization of the synthesis conditions of the nanoshells as well as the investigation of their biocompatibility are currently under way. Thus, our findings have shown the successful synthesis of a gold-silver alloy shell on composite magnetite nanocores. In addition, these nanosystems hold great promise for medical applications since based on their composition, the nanoparticles should be able to overcome corrosion and toxicity issues as well as aggregation. Furthermore, the magnetic properties of the nanocores and the thickness of the alloy shell may be systematically investigated and exploited. Complete structural characterizations and measurements of these relevant physical properties of these novel nanoshells are currently underway as well as the evaluation of the nanoshells in biological assays including in-depth cytotoxicity studies and ablation studies in various cell lines.

More details of the sample characterization including results from TEM, UV-Vis-NIR, and *M*(*T*) measurements are given in "Figure S4 in Additional file 1."

## Competing interests

The authors declare that they have no competing interests.

## Authors' contributions

DG participated in the study design, synthesized the nanoparticles, performed the physical characterization (XRD, ICP-AES, TEM, SEM/EDS, UV) of the nanoparticles, and drafted the manuscript. LC prepared MRI samples and assisted in the analysis of the MRI data. CK designed MRI studies and analyzed MRI data. AB designed magnetization studies and analyzed magnetization data. JP assisted in the synthesis and characterization of the nanoparticles. RB and JY assisted in the study design and interpretation of the UV data. MB conceived of the study, participated in the design and coordination of the study, and assisted in the drafting of the manuscript.

## Supplementary Material

Additional file 1 **Figure S1**. TEM images of gold-silver alloy (Au:Ag = 5:1) nanoshells deposited on the gold-seeded functionalized Gd:Fe_3_O_4 _nanocores. Images A-C indicate dispersed nanoparticles covered with a lighter gold-silver alloy nanoshell. **Figure S2**. TEM images of gold-silver alloy (Au:Ag = 1:1) nanoshells deposited on the gold-seeded functionalized Gd:Fe_3_O_4 _nanocores with an average size diameter of 8.2 ± 0.4 nm **(A) **and 9.7 ± 0.4 nm **(B)**. Images A and B indicate less dispersed nanoparticles than in the case of using a Au:Ag ratio of 5:1. No gold-silver alloy nanoshells are visible. **Figure S3**. UV-Vis-NIR spectra of gold-silver alloy nanoshells (Au:Ag = 1:1) deposited on gold-seeded functionalized Gd:Fe_3_O_4 _nanocores for 1 **(A)**,7 **(B) **and 14 days **(C) **reaction time. **Figure S4**. Temperature dependence of the ZFC and FC magnetization of the Au-Ag alloy (1:1) nanoshell sample in an external field of 200 Oe. Click here for file
