# Supplementary material for: Gold-silver alloy nanoshells: a new candidate for nanotherapeutics and diagnostics
Source: Nanoscale Res Lett. 2011 Oct 13;6(1):554. doi: 10.1186/1556-276X-6-554 (PMC3212091; doi:10.1186/1556-276X-6-554)
Supplement: Additional file 1 — Figure S1. TEM images of gold-silver alloy (Au:Ag = 5:1) nanoshells deposited on the gold-seeded functionalized Gd:Fe3O4 nanocores. Images A-C indicate dispersed nanoparticles covered with a lighter gold-silver alloy nanoshell. Figure S2. TEM images of gold-silver alloy (Au:Ag = 1:1) nanoshells deposited on the gold-seeded functionalized Gd:Fe3O4 nanocores with an average size diameter of 8.2 ± 0.4 nm (A) and 9.7 ± 0.4 nm (B). Images A and B indicate less dispersed nanoparticles than in the case of using a Au:Ag ratio of 5:1. No gold-silver alloy nanoshells are visible. Figure S3. UV-Vis-NIR spectra of gold-silver alloy nanoshells (Au:Ag = 1:1) deposited on gold-seeded functionalized Gd:Fe3O4 nanocores for 1 (A),7 (B) and 14 days (C) reaction time. Figure S4. Temperature dependence of the ZFC and FC magnetization of the Au-Ag alloy (1:1) nanoshell sample in an external field of 200 Oe. [file 1556-276X-6-554-S1.DOC]

SUPPORTING INFORMATION

**Gold-Silver Alloy Nanoshells: A New Candidate for Nanotherapeutics and Diagnostics**

Dana E. Gheorghe^1^, Lili Cui^1^, Christof Karmonik^2^, Audrius Brazdeikis^3^, Jose M. Penaloza^1^, Joseph K. Young^4^, Rebekah A. Drezek^4,5^, and Malavosklish Bikram^1,*^

^1^Department of Pharmacological & Pharmaceutical Sciences, College of Pharmacy, University of Houston, Texas Medical Center Campus, 1441 Moursund St., Houston, TX, 77030, USA

^2^The Methodist Hospital, 6565 Fannin, Houston, TX, 77030, USA

^3^Department of Physics and Texas Center for Superconductivity, University of Houston, 4800 Calhoun Road, Houston, Texas 77004

^4^Department of Electrical and Computer Engineering, Rice University, 6100 Main Street, MS-366, Houston, TX 77005, USA

^5^Department of Bioengineering, Rice University, 6100 Main Street, MS-142, Houston, TX 77005, USA

*Corresponding author

E-mail: mbikram@Central.UH.EDU

*Corresponding author: mbikram@Central.UH.EDU. Phone: +1 713 795 8317. Fax: +1 713 795 8305.


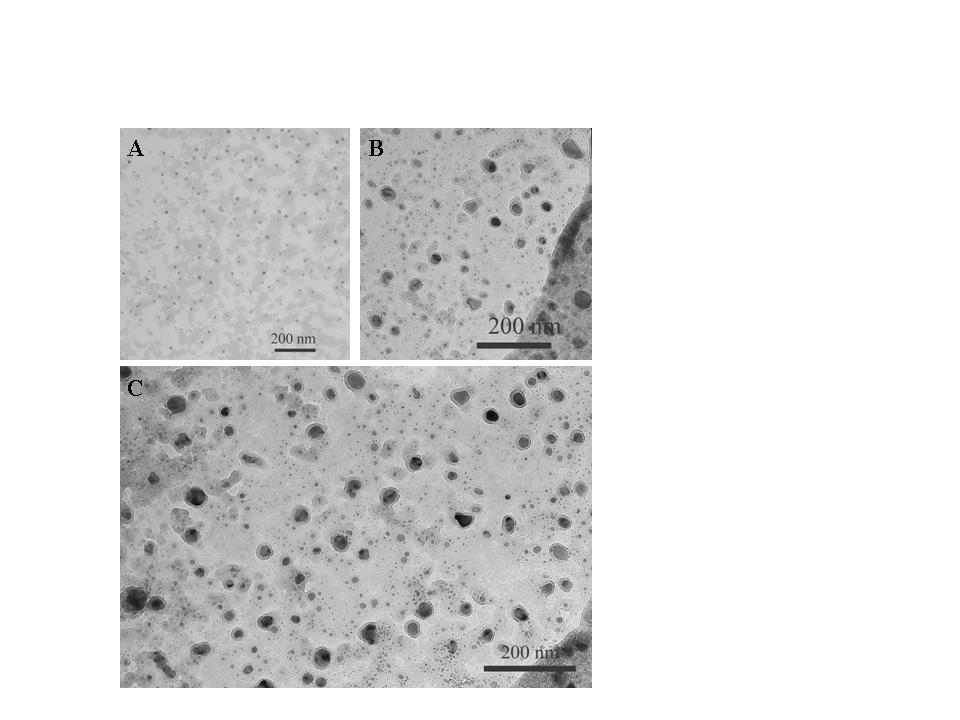


**Figure S1.** TEM images of gold-silver alloy (Au:Ag=5:1) nanoshells deposited on the gold seeded functionalized Gd:Fe_3_O_4_ nanocores. Images A-C indicate dispersed nanoparticles covered with a lighter gold-silver alloy nanoshell.

**
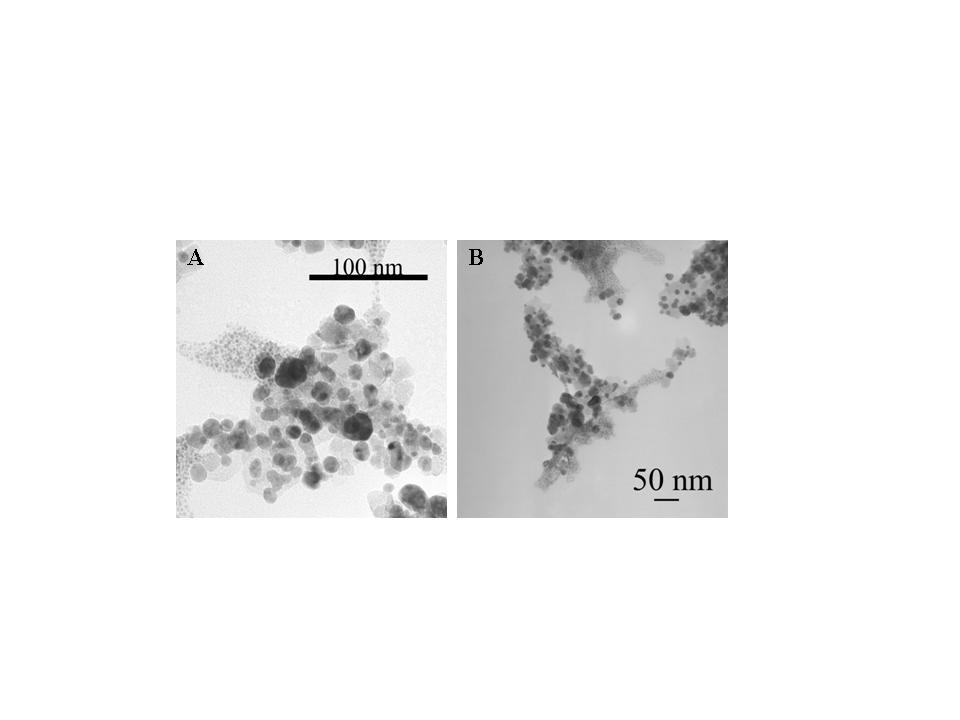
**

**Figure S2.** TEM images of gold-silver alloy (Au:Ag=1:1) nanoshells deposited on the gold seeded functionalized Gd:Fe_3_O_4_ nanocores with an average size diameter of 8.2 ± 0.4 nm (A) and 9.7 ± 0.4 nm (B). Images A and B indicate less dispersed nanoparticles than in the case of using a Au:Ag ratio of 5:1. No gold-silver alloy nanoshells are visible.

**Figure S3.** UV-Vis-NIR spectra of gold-silver alloy nanoshells (Au:Ag=1:1) deposited on gold-seeded functionalized Gd:Fe_3_O_4_ nanocores for 1 (A),7 (B) and 14 days (C) reaction time.


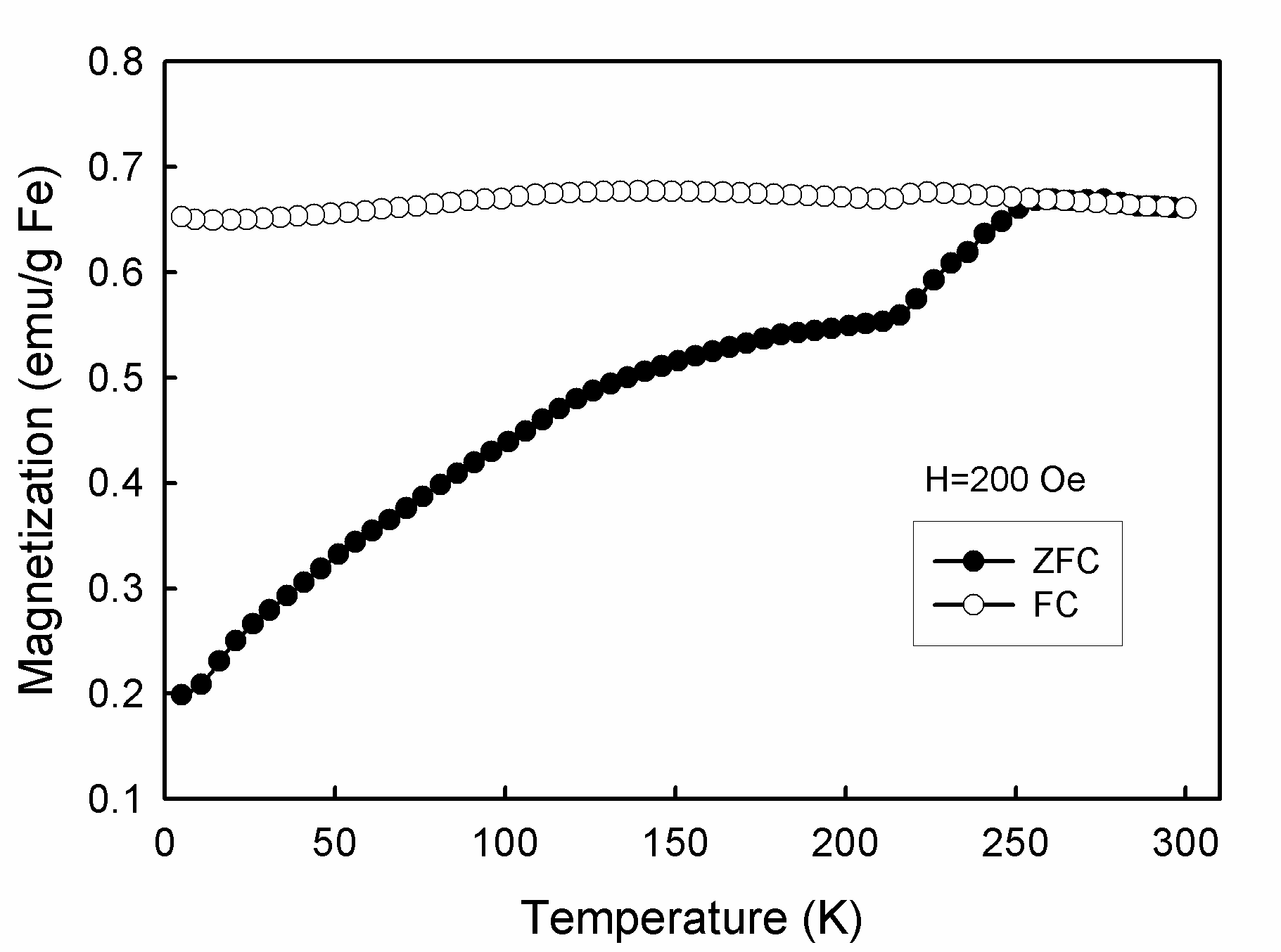


**Figure S4.** Temperature dependence of the zero-field-cooled (ZFC) and field-cooled (FC) magnetization of the Au-Ag alloy (1:1) nanoshell sample in an external field of 200 Oe.
